# 2D Geometry Predicts Perceived Visual Curvature in Context-Free Viewing

**DOI:** 10.1155/2015/708759

**Published:** 2015-08-05

**Authors:** Birgitta Dresp-Langley

**Affiliations:** ICube, UMR 7357, CNRS, Université de Strasbourg, 2 rue Boussingault, 67000 Strasbourg, France

## Abstract

Planar geometry was exploited for the computation of symmetric visual curves in the image plane, with consistent variations in local parameters such as* sagitta*,* chordlength*, and the curves' height-to-width ratio, an indicator of the visual area covered by the curve, also called aspect ratio. Image representations of single curves (no local image context) were presented to human observers to measure their visual sensation of curvature magnitude elicited by a given curve. Nonlinear regression analysis was performed on both the individual and the average data using two types of model: (1) a power function where *y* (sensation) tends towards infinity as a function of *x* (stimulus input), most frequently used to model sensory scaling data for sensory continua, and (2) an “exponential rise to maximum” function, which converges towards an asymptotically stable level of *y* as a function of *x*. Both models provide satisfactory fits to subjective curvature magnitude as a function of the height-to-width ratio of single curves. The findings are consistent with an in-built sensitivity of the human visual system to local curve geometry, a potentially essential ground condition for the perception of concave and convex objects in the real world.

## 1. Introduction

**Figure 1:**
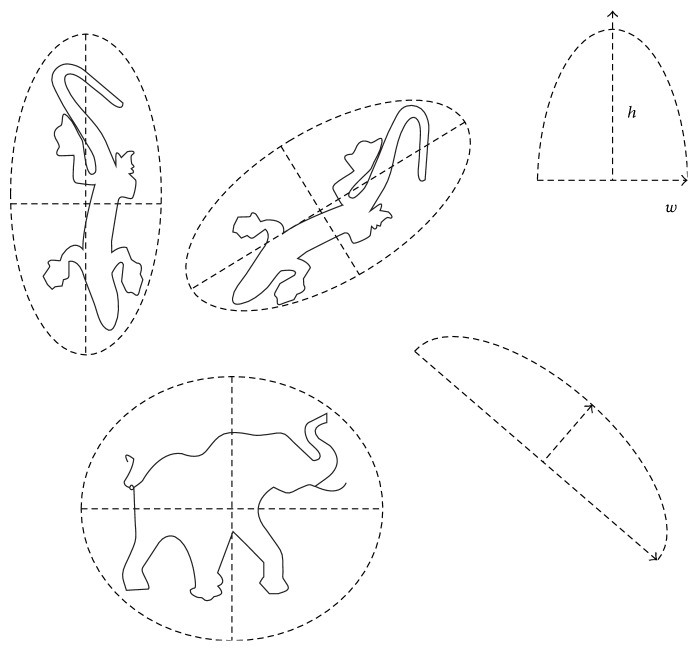
Most objects represented by line contours in the two-dimensional image plane cover a space that roughly corresponds to the shape of an ellipse. The receptive field structures of visual cortical detectors in the primate brain also cover areas which are roughly elliptic. The height-to-with ratio (*h*/*w*), sometimes also called* aspect ratio*, of 2D shapes is a geometric parameter relative to the visual area covered by a curve.

**Figure 2:**
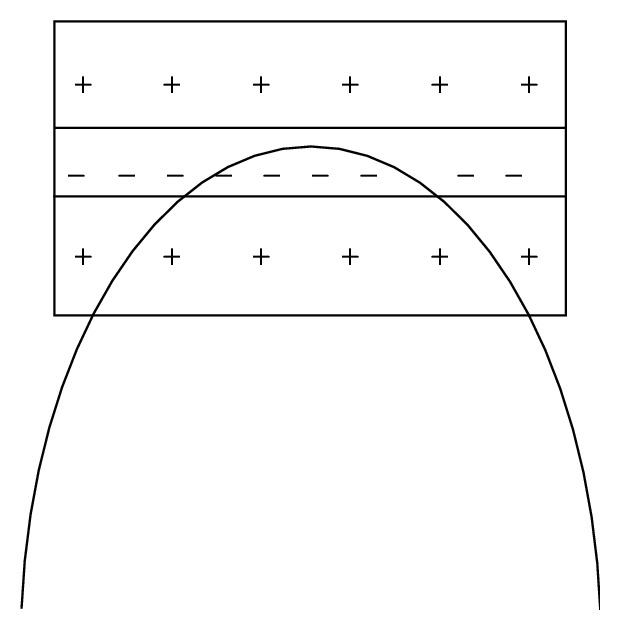
Curvature selective visual cortical neurons of one and the same coding population respond optimally to deviations from a single straight line on the basis of functionally identified receptive field properties, which include contrast sensitivity and selectivity to local contrast signs (shown here schematically, for illustration). A multitude of such curvature mechanisms operate in parallel in the primates' visual brain.

The question whether the human brain may have an in-built sense of geometry has led to the emergence of new approaches to visual cognition (e.g., [1]). Since our visual environment abounds with curved shapes and features, the question whether our brain is sensitive to the geometric properties of visual curves comes to mind. Local two-dimensional (2D) curvature is a highly informative visual cue for global shape perception, object recognition, and image interpretation (e.g., [2–8]). Nonconscious brain representations of local stimulus geometry may enable conscious knowledge about object properties and associations between specific two-dimensional projections and their correlated three-dimensional structures in the real world (e.g., [1, 9–14]). Objects represented in the two-dimensional image plane cover spaces with a roughly elliptic geometry (Figure 1). The receptive field structures of visual cortical neurons (curvature detectors) in the primate brain, sensitive to local 2D properties of curve stimuli, are also roughly elliptic (e.g., [15–17]). Global shape representation is enabled by local stimulus biases favouring symmetry and other 2D structural regularities [14, 18]. Neurons of the same coding population, responding optimally to deviations from a single straight line (Figure 2), constitute a whole curvature-processing network in the primate brain [19].

At the level of neural processing, current models of curvature coding based on functional properties of visual cortical cell populations in the primate brain postulate curvature mechanisms that operate in parallel [19]. They ensure the processing of local input from over the visual field and encode curvature for all orientations and for a range of curvature amplitudes. Curvature mechanisms are conceived as the combination of the responses of several coding populations, with their receptive fields arranged along several such curved lines in complex images. Neural response activity is optimal when the line contour matches locations and orientations to which the neurons of a given population are selective. Perceived curvature would then result from the interaction of mechanisms that operate on spatially local contour curvature signals with higher level processes that serve to establish global shape [8, 20]. What has remained unclear is which critical information contained in a curve stimulus produces the optimal neural response, and whether this suffices to account for optimal curvature perception.

**Figure 3:**
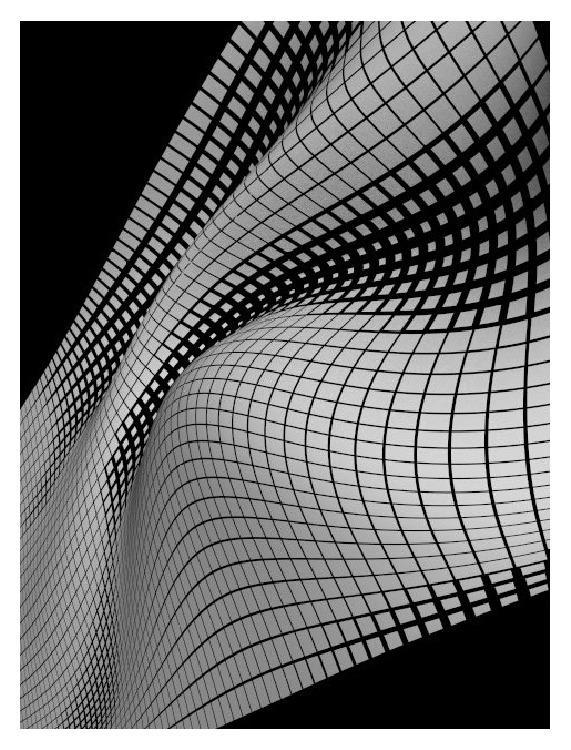
In complex images, local curvature permits generating strong three-dimensional shape effects. Curvature processing can be made easier or rendered more difficult when a local curve (target) is embedded in such a complex scene context of multiple curves. Contextual effects of this kind are explained in terms of long-range neural interactions (see [23], for review).

At the behavioral level, curvature processing depends on the visual context of the curved target within the scene context (Figure 3). The visual salience of curves changes with the direction, the magnitude, and the immediate context of the stimuli, which has important implications for the development of visual interface technologies [7]. Perceptual interactions between curves have been explained in terms of influences from large-scale neural averaging occurring in high-level image processing (e.g., [7, 21]), but this explanation does not help understand what actually determines our perception of a curve. Thus, to find out which local information in a curve critically determines curvature perception, we need to investigate visual sensations in response to single, preferably symmetric curves under conditions that are as context-free as possible (i.e., no immediate image context). Previous research has shown that local curvature signals are strongest when no immediate image context is given [21].

The goal of this study here was to clarify which local information in a curve critically determines the strength of visual sensations of curvature in response to single curves with consistently varying geometric properties under conditions of context-free viewing. A psychophysical scaling procedure is used to bring the issue whether a particular local geometric property accounts for the perceived strength of the curves to the fore. In psychophysics there are several types of measurement (see [22], for an up-to-date review). One relies on experimental protocols that allow manipulations of physical variables to be reflected back from an experimental participant into the physical world. The participant's response to a stimulus is measured by counting, for example, the proportion of hits and false alarms as in signal detection theory, or the time to respond to a stimulus (response time). The level of some physical variable, like sound or light intensity, required to reach a certain performance criterion (detection or discrimination threshold) may also be measured. Another type of measurement, the one used in this study here, involves participants' reporting directly on the magnitude of a sensory or other subjective experiences such as the magnitude of brightness, darkness, or curvature (as here) perceived in a single stimulus. To this effect a typical but informal scale (most often a category scale from 1 to 10 as here in this study) is used on sample populations of three to ten observers.

## 2. Material and Methods

Computations based on strictly local curve geometry were implemented to generate a whole set of single images of visual curves with variable symmetric curvature in the two-dimensional image plane. Images of arcs, corresponding to lower and upper halves of ellipses, were derived mathematically through planar projection by affinity with circles (Figure 5). This computation permits generating curved lines in the 2D plane (using AUTOCAD or equivalent software) with consistent variations in 2D parameters (Figure 5) for* sagitta*,* chordlength*, and* height-to-width ratio* (*h*/*w*), an index which conveys spatial information relative to the visual area covered by a curve [2, 3]. The experiments were conducted in accordance with the Declaration of Helsinki (1964). Visual images for the experiments were generated in AUTOCAD. Statistical analyses of the visual data were performed using SYSTAT.

### 2.1. Curve Computation

Elliptic arcs of planar ellipses were computed using projective geometry and the principle of transformation by affinity with concentric circles (Figure 5), a relatively simple procedure for computational image generation. To explain how ellipses are obtained in this way, it is useful to recall some of the properties of concentric circles, which share the same centre. In the two-dimensional plane, a so-called principal circle with centre 0  (*C*_0,*a*_) is defined in terms of

(1) $$\begin{array}{c} R^{2}\left(\;C_{0,a}\;\right)={\left(\;x\;\right)}^{2}+{\left(\;y\;\right)}^{2}, \end{array}$$

where *R* is the radius of the circle and *x* and *y* the two-dimensional spatial coordinates of the points falling on its perimeter. A second concentric circle is obtained from the first one by

(2) $$\begin{array}{c} R^{2}\left(\;C_{0,b}\;\right)={\left(\;x+\delta x\;\right)}^{2}+{\left(\;y+\delta y\;\right)}^{2} \end{array}$$

or

(3) $$\begin{array}{c} R^{2}\left(\;C_{0,b}\;\right)={\left(\;x-\delta x\;\right)}^{2}+{\left(\;y-\delta y\;\right)}^{2}. \end{array}$$

Ellipses as projected images of concentric circles (Figure 2) may be defined in terms of

(4) $$\begin{array}{c} \left(\;x,y\;\right)=\left(\;bx,ay\;\right) \end{array}$$

of the principal circle *C*_(0,*a*)_ and

(5) $$\begin{array}{c} \left(\;x,y\;\right)=\left(\;\left(\;ab\;\right)x,y\;\right) \end{array}$$

of the secondary circle *C*_(0,*b*)_. This transform is sometimes referred to as a particular case of Newton's transform. In the two-dimensional plane, an ellipse (*E*) is thus defined in terms of

(6) $$\begin{array}{c} E=x^{2}a^{2}+y^{2}b^{2}=1, \end{array}$$

with axes *a* and *b* are the axes of symmetry intersecting at the ellipse's centre. The larger axis of the two is referred to as the major and the smaller as the minor. The majors and the minors are directly linked to the* sagitta*, or maximum height (*h*), and the* chordlength*, or width (*w*) of elliptic arcs (Table 1). The curves were presented as individual images of white curves on dark backgrounds (Figure 3). Presentations were generated on an IBM computer (Pentium III) equipped with a standard colour screen with a display resolution of 1024 × 768 pixels. The curves, with “positive” (upward) and “negative” (downward) curvature in the two-dimensional plane, corresponded to 22 elliptic arcs, derived from concentric circles with varying diameter through planar projection by affinity as described here above. The luminance of the bright curves was 40 cd/m^2^, measured with a standard photometer (Cambridge Research Systems), equipped with software for calibrating grey levels (R–G–B combinations) of a computer screen. The dark background of the screen on which the curves were presented had a constant luminance (2 cd/m^2^).

### 2.2. Subjects

Nine observers (five women and four men), all of whom are graduate students in neuroscience at the University of Montpellier, aged between 24 and 26 and with normal or corrected-to-normal vision participated in the experiments. All were naive to the purpose of the study. Individual experimental sessions were run, with the individual seated comfortably in a semidark room in front of a computer screen.

### 2.3. Procedure

**Figure 4:**
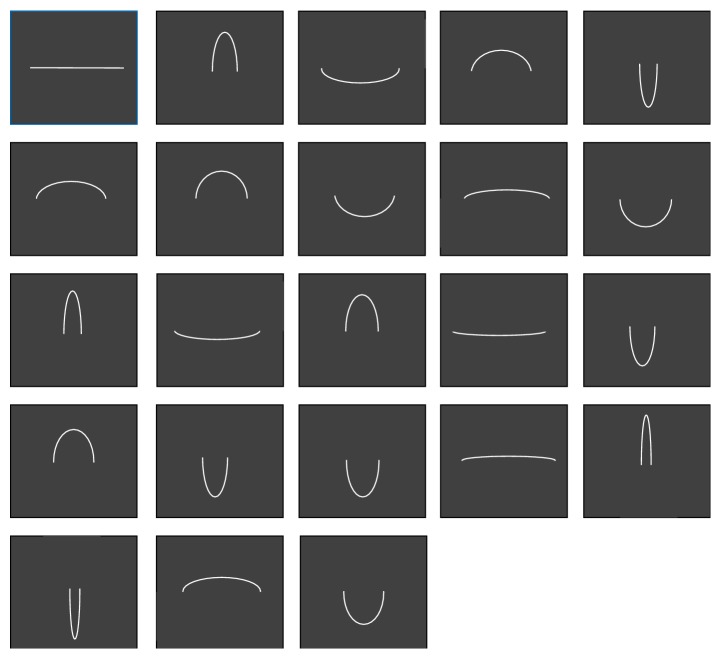
In this study here, image context effects were excluded by presenting images of a single curve, one after another in random order. In this way, curvature operators from only one, not many different coding channels, were stimulated on a given experimental trial.

**Figure 5:**
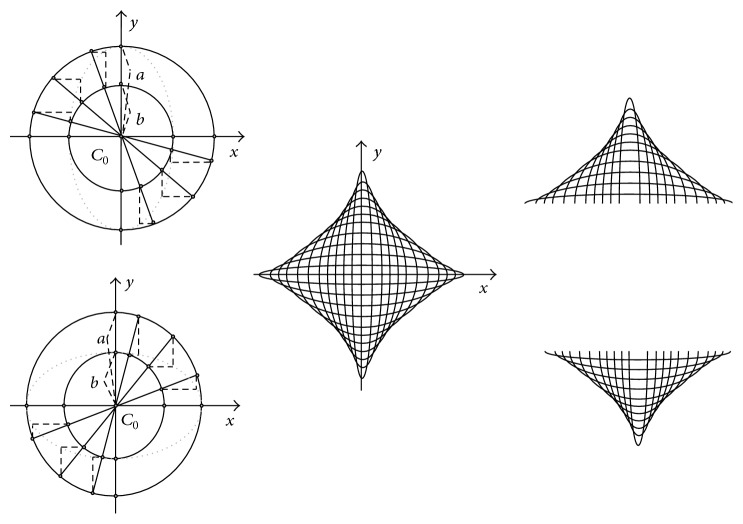
Vertically and horizontally oriented ellipses in the two-dimensional plane can be obtained from concentric circles through a geometric transform called planar projection by affinity. In Cartesian space, an ellipse may be defined as the projected image of two concentric circles. In the two examples given here, images (*x*, *y*) = (*b* ^*x*^, *a* ^*y*^) of the principal circle *C*_(0,*a*)_ and images (*x*, *y*) = ((*a*/*b*)*x*, *y*) of the secondary circle *C*_(0,*b*)_ generate ellipses through planar projection by affinity with the two circles. In our study, upward oriented and downward oriented arcs of eleven such ellipses, derived from concentric circles with varying diameter, were generated.

Observers were told that they were going to view a series of curves, one at a time, and were asked to type a number between 0 and 10 that was to reflect the magnitude of curvature that came up on the screen in a given trial. The curves (Figure 4) were presented in random order, and for each observer, a different random sequence of stimuli was generated. A perfectly straight, white line with zero curvature was shown on the screen at the beginning of the experimental trials to clarify the visual standard for “zero curvature.” This control condition was repeated five times during a session, with control trials randomly positioned within a sequence of test trials. This allowed for making sure that subjects consistently replied “0” to the zero curvature stimulus. The duration of an image presentation was one second, and observers were encouraged to give their rating as rapidly as possible. Typing the “enter” key triggered the presentation of the next stimulus. Each curve was shown twice within a single individual session of trials.

## 3. Results

**Figure 6:**
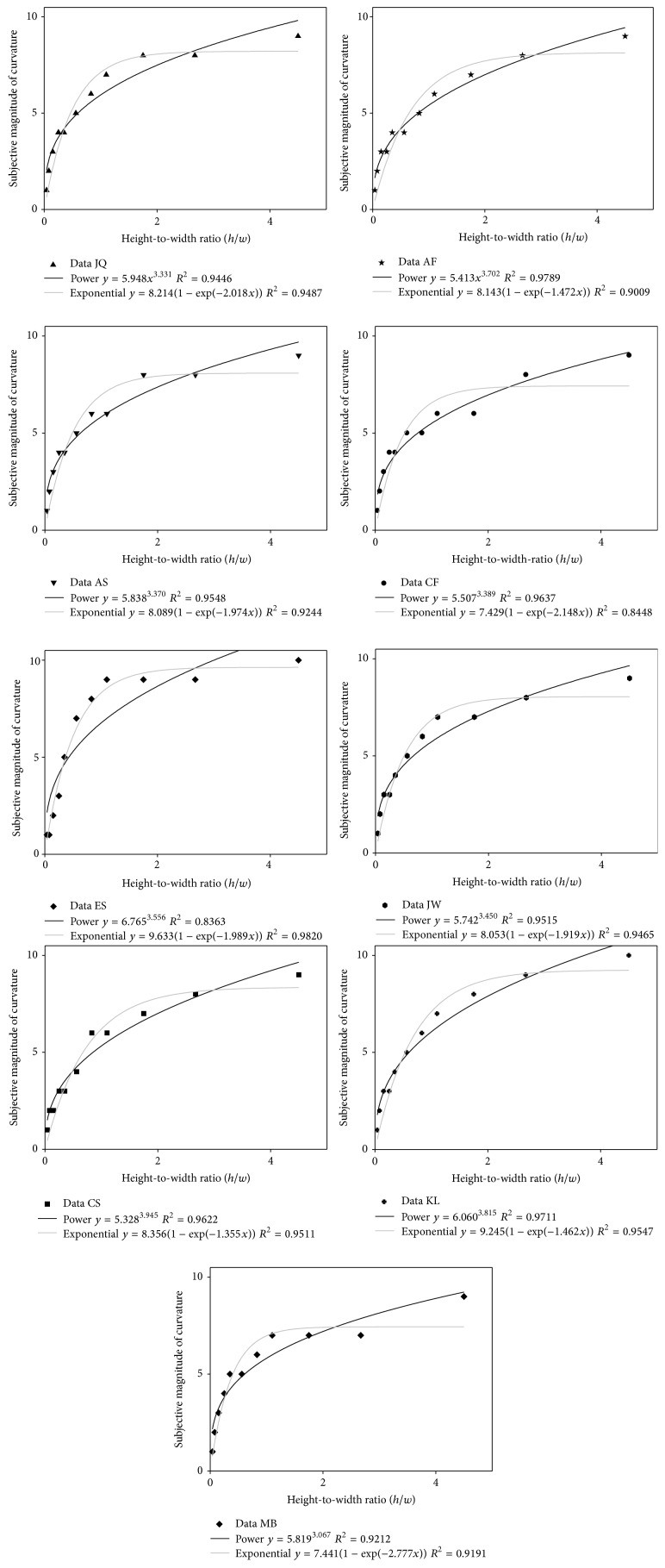
The individual psychometric functions are shown here. Subjective magnitudes of visual curvature are plotted as a function of the* height-to-width ratio* of the curves.

Individual psychometric functions of subjective curve magnitude as a function of the curves' height-to-width ratio were plotted. “Positive” and “negative” curves with identical height-to-width ratios produced identical or very similar magnitudes, as could be expected from previous data reported by Dresp et al. [5], and these data were therefore averaged. The individual results, averaged over the curve orientation factor, are shown in the graphs in Figure 6. Nonlinear regression analysis was performed on both the individual and the average data using two types of model: (1) a power function where *y* (sensation) tends toward infinity as a function of *x* (stimulus input), most frequently used to model sensory scaling data for sensory continua, and (2) an “exponential rise to maximum” function, which converges towards an asymptotically stable level of *y* as a function of *x*. The exponential-rise-to-maximum function thus levels out flat without progression toward infinity. The exponential-rise-to-maximum function is written in terms of

(7) $$\begin{array}{c} y=a\left(\;1-\exp\left(\;-bx\;\right)\;\right). \end{array}$$

The power function is expressed in terms of

(8) $$\begin{array}{c} y=ax^{b}. \end{array}$$

**Figure 7:**
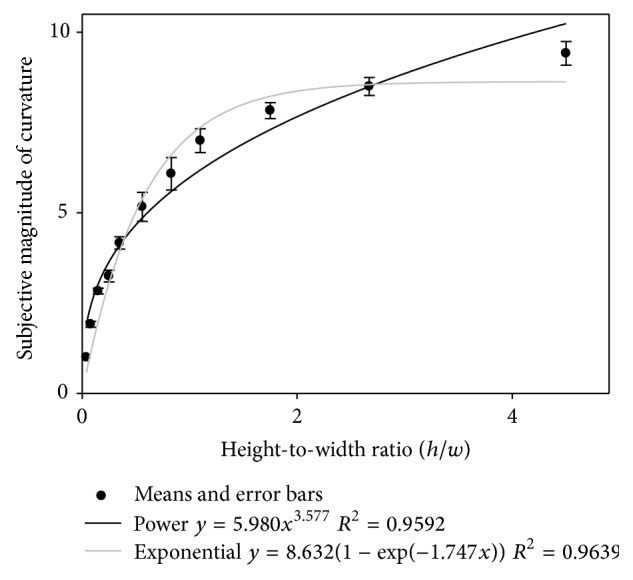
The psychometric function describing the average data is shown here, with errors bars. Subjective magnitudes of visual curvature, with power fit and exponential-rise-to-maximum fit, are plotted as a function of the* height-to-width ratio* of the curves.

The goodness of fit of these models was assessed on the basis of nonlinear regression analysis. The numerical parameter values for *a*, *b*, the regression coefficient *R* ^2^, and the associated probability limits (*p*) for each type of fit are summarized in Tables 2(a) and 2(b). The results from the nonlinear regression analyses show that the exponential-rise-to-maximum function and the power function produce reasonably good model fits to the individual data. Fits to the average data (shown in Figure 7, with error bars) confirm these conclusions.

## 4. Discussion

The visual magnitude of curvature in response to images of single curves without other local image contexts consistently increases with the* aspect ratio* of the curves, a two-dimensional geometry based shape index (e.g., [11, 18]). Context-free viewing is potentially critical to this finding. When a curved target is presented together with other curves in a complex scene context, the visual processing of the target is influenced by the context and becomes more difficult to predict. Such contextual effects are likely to be due to influences from large-scale neural interactions (see [23], for review) in networks of cortical operators, functionally identified in the primate brain [19]. In this study here, context effects on the curvature ratings can be excluded given that curvature operators from different coding populations were not stimulated. Instead, we may assume locally independent curvature processing, where the effect of a single curvature signal at a given trial, presented without any other image context here (no local or global interactions), can be directly associated with the curvature estimate it produced. Previous psychophysical studies of visual curvature coding [21] had shown that a local curvature signal is strongest in brief viewing and in response to images with a single symmetric curve. Symmetry of the curves is probably another important factor [14]. The mechanisms which explain why symmetry helps reduce uncertainty in visual processing are unclear. It has been suggested that all visual 3D interpretations consistent with a single 2D image would be mirror symmetric, which could imply that our visual brain has evolved toward an optimal sensitivity to symmetrical stimulus input. Also, the process that leads to 3D shape recovery often depends on the* aspect ratio*, or height-to-width ratio, of shapes, and the visual system appears to compute this parameter on the basis of criteria for minimum surface area and maximal planarity of contours [11, 18]. The existence of visual mechanisms that rely on local 2D shape geometry to recover a 3D shape interpretation makes good sense and confirms conclusions from earlier studies (e.g., [8, 9, 24]). The idea of local mechanisms for global shape recovery is consistent with the fact that shape recognition is viewpoint independent as far as the projected image does not change substantially under small or moderate changes in the viewing direction of the shape (e.g., [14]). Sensitivity to planar geometry thus appears as a potentially important aspect of brain processing, essential for generating the most likely shape interpretation on the basis of relatively simple computations. The findings from this study here indicate that the human perceptual system is definitely sensitive to the local geometry of curve stimuli. Whether this sensitivity is in-built or learnt remains to be clarified in experiments testing its ontogenetic development (cf. [1]). Whether the power law or the exponential rise to maximum law should be preferred to model the findings depends on conceptual issues relative to sensory continua (cf. [22] for a recent review) which are beyond the scope of this paper and potentially irrelevant, as both functions are shown to produce reasonably good fits.

## 5. Conclusions

Curved objects represented in the 2D image plane can be computed on the basis of a very simple mathematical transform based on planar shape geometry. These computations yield consistent variations in a limited number of critical curve parameters. Power and exponential rise to maximum models adequately account for curvature magnitude scaled by human observers as a function of local curve parameters relative to the two-dimensional visual area covered by the curve, the* height-to-width ratio*, showing that the visual magnitude of curvature in planar images can be consistently linked to this local parameter. The conclusions lend support to theories of geometry based brain representations for the perception or recovery of complex shape information from two-dimensional images (e.g., [1, 10, 13, 18]).

## Figures and Tables

**Table 1:** Values in centimetres (on the screen) for *sagitta* or maximum height (*h*), *chordlength* or width (*w*), and aspect ratio (*h*/*w*) of the curves, presented here as individual images of white visual contours on dark backgrounds.

*Sagitta *	*Chordlength *	Aspect ratio

2 cm	9 cm	4.50
3 cm	8.5 cm	2.83
4.5 cm	8 cm	1.77
6.5 cm	7.5 cm	1.15
8 cm	7 cm	0.87
8.5 cm	6 cm	0.70
9 cm	5 cm	0.50
10 cm	4 cm	0.40
12 cm	3 cm	0.25
14 cm	2 cm	0.14
18 cm	1 cm	0.05

**Table:** (a) Exponential

*a*	*b*	*R* ^2^
8.21	−2.01	0.94
8.14	−1.47	0.90
8.09	−1.97	0.92
7.43	−2.14	0.84
8.36	−1.35	0.95
9.63	−1.99	0.98
8.05	−1.92	0.95
9.24	−1.46	0.95
7.44	−2.77	0.91

**Table:** (b) Power

*a*	*b*	*R* ^2^
5.94	3.33	0.94
5.41	3.70	0.98
5.83	3.37	0.95
5.50	3.39	0.96
5.33	3.94	0.96
6.76	3.56	0.84
5.74	3.45	0.95
6.06	3.81	0.97
5.82	3.06	0.92
